# A large data resource of genomic copy number variation across neurodevelopmental disorders

**DOI:** 10.1038/s41525-019-0098-3

**Published:** 2019-10-07

**Authors:** Mehdi Zarrei, Christie L. Burton, Worrawat Engchuan, Edwin J. Young, Edward J. Higginbotham, Jeffrey R. MacDonald, Brett Trost, Ada J. S. Chan, Susan Walker, Sylvia Lamoureux, Tracy Heung, Bahareh A. Mojarad, Barbara Kellam, Tara Paton, Muhammad Faheem, Karin Miron, Chao Lu, Ting Wang, Kozue Samler, Xiaolin Wang, Gregory Costain, Ny Hoang, Giovanna Pellecchia, John Wei, Rohan V. Patel, Bhooma Thiruvahindrapuram, Maian Roifman, Daniele Merico, Tara Goodale, Irene Drmic, Marsha Speevak, Jennifer L. Howe, Ryan K. C. Yuen, Janet A. Buchanan, Jacob A. S. Vorstman, Christian R. Marshall, Richard F. Wintle, David R. Rosenberg, Gregory L. Hanna, Marc Woodbury-Smith, Cheryl Cytrynbaum, Lonnie Zwaigenbaum, Mayada Elsabbagh, Janine Flanagan, Bridget A. Fernandez, Melissa T. Carter, Peter Szatmari, Wendy Roberts, Jason Lerch, Xudong Liu, Rob Nicolson, Stelios Georgiades, Rosanna Weksberg, Paul D. Arnold, Anne S. Bassett, Jennifer Crosbie, Russell Schachar, Dimitri J. Stavropoulos, Evdokia Anagnostou, Stephen W. Scherer

**Affiliations:** 1 The Centre for Applied Genomics, The Hospital for Sick Children, Toronto, ON Canada; 2 Program in Genetics and Genome Biology, The Hospital for Sick Children, Toronto, ON Canada; 3 Neurosciences and Mental Health Program, The Hospital for Sick Children, Toronto, ON Canada; 4 Genome Diagnostics, Department of Paediatric Laboratory Medicine, The Hospital for Sick Children, Toronto, ON Canada; 5 Department of Molecular Genetics, University of Toronto, Toronto, ON Canada; 6 Clinical Genetics Research Program, Centre for Addiction and Mental Health, Toronto, ON Canada; 7 Division of Clinical and Metabolic Genetics, The Hospital for Sick Children, Toronto, ON Canada; 8 Medical Genetics Residency Training Program, University of Toronto, Toronto, ON Canada; 9 Department of Genetic Counselling, The Hospital for Sick Children, Toronto, ON Canada; 10 The Prenatal Diagnosis and Medical Genetics Program, Department of Obstetrics and Gynecology, Mount Sinai Hospital, Toronto, ON Canada; 11 Department of Paediatrics, University of Toronto, Toronto, ON Canada; 12 Deep Genomics Inc., Toronto, ON Canada; 13 Hamilton Health Sciences, Ron Joyce Children’s Health Centre, Hamilton, On Canada; 14 Trillium Health Partners Credit Valley Site, Mississauga, Ontario Canada; 15 Department of Psychiatry, University of Toronto, Toronto, ON Canada; 16 Autism Research Unit, The Hospital for Sick Children, Toronto, ON Canada; 17 Laboratory Medicine and Pathobiology, University of Toronto, Toronto, ON Canada; 18 Department of Psychiatry and Behavioral Neurosciences, Wayne State University, Detroit, MI USA; 19 The Children’s Hospital of Michigan, Detroit, MI United States; 20 Department of Psychiatry, University of Michigan, Ann Arbor, MI USA; 21 Institute of Neuroscience, Newcastle University, Newcastle upon Tyne, UK; 22 Dalla Lana School of Public Health and the Department of Family and Community Medicine, University of Toronto, Toronto, ON Canada; 23 Department of Pediatrics, University of Alberta, Edmonton, AB Canada; 24 Montreal Neurological Institute, McGill University, Montreal, QC Canada; 25 Discipline of Genetics, Faculty of Medicine, Memorial University of Newfoundland, St. John’s, NL Canada; 26 Regional Genetics Program, The Children’s Hospital of Eastern Ontario, Ottawa, ON Canada; 27 Centre for Addiction and Mental Health, Toronto, ON Canada; 28 Department of Psychiatry, The Hospital for Sick Children, Toronto, ON Canada; 29 Mouse Imaging Centre, Hospital for Sick Children, Toronto, ON Canada; 30 Department of Medical Biophysics, The University of Toronto, Toronto, ON Canada; 31 Department of Psychiatry, Queen’s University, Kinston, ON Canada; 32 Children’s Health Research Institute, London, ON Canada; 33 Western University, London, ON Canada; 34 Department of Psychiatry and Behavioural Neurosciences, McMaster University, Hamilton, ON Canada; 35 Mathison Centre for Mental Health Research and Education, University of Calgary, Calgary, AB Canada; 36 Departments of Psychiatry and Medical Genetics, Cumming School of Medicine, University of Calgary, Calgary, AB Canada; 37 The Dalglish Family 22q Clinic, Toronto General Hospital, Toronto, ON Canada; 38 Institute of Medical Science, University of Toronto, Toronto, ON Canada; 39 Holland Bloorview Kids Rehabilitation Hospital, University of Toronto, Toronto, ON Canada; 40 Department of Molecular Genetics and McLaughlin Centre, University of Toronto, Toronto, ON Canada

**Keywords:** Molecular medicine, Pathogenesis, Neurodevelopmental disorders

## Abstract

Copy number variations (CNVs) are implicated across many neurodevelopmental disorders (NDDs) and contribute to their shared genetic etiology. Multiple studies have attempted to identify shared etiology among NDDs, but this is the first genome-wide CNV analysis across autism spectrum disorder (ASD), attention deficit hyperactivity disorder (ADHD), schizophrenia (SCZ), and obsessive-compulsive disorder (OCD) at once. Using microarray (Affymetrix CytoScan HD), we genotyped 2,691 subjects diagnosed with an NDD (204 SCZ, 1,838 ASD, 427 ADHD and 222 OCD) and 1,769 family members, mainly parents. We identified rare CNVs, defined as those found in <0.1% of 10,851 population control samples. We found clinically relevant CNVs (broadly defined) in 284 (10.5%) of total subjects, including 22 (10.8%) among subjects with SCZ, 209 (11.4%) with ASD, 40 (9.4%) with ADHD, and 13 (5.6%) with OCD. Among all NDD subjects, we identified 17 (0.63%) with aneuploidies and 115 (4.3%) with known genomic disorder variants. We searched further for genes impacted by different CNVs in multiple disorders. Examples of NDD-associated genes linked across more than one disorder (listed in order of occurrence frequency) are *NRXN1*, *SEH1L*, *LDLRAD4*, *GNAL*, *GNG13*, *MKRN1*, *DCTN2, KNDC1*, *PCMTD2*, *KIF5A*, *SYNM*, and long non-coding RNAs: *AK127244* and *PTCHD1-AS*. We demonstrated that CNVs impacting the same genes could potentially contribute to the etiology of multiple NDDs. The CNVs identified will serve as a useful resource for both research and diagnostic laboratories for prioritization of variants.

## Introduction

Genomic copy number variations (CNVs) are structural variations that involve deletions and/or duplications of segments of DNA. Their impact is not necessarily harmful, but loss, increase, or disruption of genes is often associated with, and can underlie human disease, including neurodevelopmental disorders (NDDs).^1–6^ Rare CNVs have been extensively studied in autism spectrum disorder (ASD),^7–9^ attention deficit hyperactivity disorder (ADHD),^10^ schizophrenia (SCZ),^11,12^ and less so, obsessive compulsive disorder (OCD).^13,14^ Various NDDs share genomic structural variations, including CNVs that perturb the same genes.^1,15^ For example, deletions or duplications affecting coding sequences of *NRXN1* or *CNTN6* have been implicated in ASD, ADHD, and SCZ.^16,17^

A shared genomic etiology across multiple disorders is supported through genome-wide association studies^18^ or analyzing small loss-of-function (LoF) variations.^19^ However, CNV analysis across disorders has been limited; e.g., ADHD and ASD,^10^ ASD and SCZ,^20^ reviews on selected well-established genomic disorders such as 16p11.2 deletions and duplications,^21^ or a meta-analysis of CNVs on a single gene (*NRXN1*^16^). To date, there has been no genome-wide study of rare CNVs identified using an identical technology, encompassing ASD, ADHD, OCD, and SCZ. An identical method for interrogating CNVs across multiple disorders increases the chance of finding rare CNVs with cross-disorder implications. These could be missed if multiple technologies with different detection sensitivities were applied.

This project was established to provide a public resource of CNV samples with NDDs mostly from the province of Ontario, Canada, all genotyped on same microarray platform: the Affymetrix CytoScan HD platform, which consists of 1.9 million copy number markers and 750,000 genotype-able single nucleotides polymorphisms. A CNV resource of control population samples was published earlier under the Ontario Population Genomics Platform.^22^

In creating this data resource, we aimed to: (i) catalogue CNVs that are clinically relevant to each of ASD, ADHD, OCD, and SCZ, and (ii) identify genes and loci with CNVs that are shared among different NDDs. Where available, we analyzed whole genome (WGS) or whole exome sequence (WES) data, in search of variants that were not detected by microarray. The relevant genotypes and CNVs are available in dbGaP (accession number phs001881.v1.p1) and dbVar (accession number nstd173), respectively.

## Results

### Sample description and detection of CNVs

We analyzed 4,460 samples ascertained for four NDDs; 2,691 (60.3%) were from individuals recruited because of the diagnosis of one of these disorders, and the rest from apparently unaffected family members (Table 1, Supplementary Table 1A). ASD, ADHD, OCD, and SCZ cases were ascertained using criteria explained previously (Supplementary Information).^8,10,13,23,24^ Childhood onset OCD is not classically considered an NDD in the International Classification of Disease (ICD-11) or Diagnostic and Statistical Manual of Mental Disorders (DSM-5), but in view of its early onset, male preponderance and association with imaging findings, we included OCD in this study as others often do. Similarly, since SCZ has both neural and genetic correlates, including some evidence of overlap or sharing genetic risk with NDDs, we considered SCZ as well. The majority of samples (68.1%) were ascertained for ASD, with the others distributed approximately equally among the other disorders. The male:female sex ratio was almost 4:1 for ASD and ADHD, 2:1 for SCZ, and ~1:1 in the pediatric OCD cases (Table 1). Different family structures were sampled in the four sub-groups: some ASD and SCZ families included multiple affected individuals; some OCD samples were in trios (i.e., affected proband and both parents; details in Table 1, Supplementary Table 1A). We did not genotype parents of cases for ADHD (Table 1, Supplementary Table 1A). We defined a high confidence set of CNVs (Supplementary Table 1B) as those identified using two different detection algorithms, as previously described.^25^ Rare variants were those with a frequency of less than 0.1% in a 10,851 subject population control sample genotyped in multiple microarray platforms, including the Affymetrix CytoScan HD.^25^ We also analyzed prioritized CNVs from our previous publications on ADHD^10^ and SCZ.^11,24^ We had information with respect to intellectual disability (ID) for some cases. ID including borderline intellect and non-verbal learning disability was comorbid with SCZ in 31/204 cases (16.9%),^11^ with ASD in 149/599 cases (24.9%), and with ADHD in 3/427 cases (0.7%). No OCD case had ID.

**Table 1:** Stratification of 4,460 samples in the cross-disorder CNV analysis

Disorder	Samples^1^	Cases^2^	Adult ^3^	Sex ratio (male/female)	Only probands^4^	Only one parent^5^	Trios^6^	Quartets^7^	Others^8^
ASD	3,034	1,838	4.0%	4.0 (1,474/366)	1,138	89	305	126	17
ADHD	427	427	0	3.7 (337/90)	427	0	0	0	0
SCZ	435^9^	204^10^	100%	2.0 (137/67)	155	36	13	0	0
OCD	564	222	0	0.9 (107/115)	49	6	167	0	0
All	4,460	2,691	10.3%	3.2 (2,055/638)	1,769	131	485	126	17

*ASD* autism spectrum disorder, *ADHD* attention deficit hyperactivity disorder, *SCZ* schizophrenia, *OCD* obsessive compulsive disorder

^1^Count of all samples that passed our stringent QC, including assessing genotype quality, removing duplicated samples or those with non-mendelian segregation implying alternative parental relationships. This includes parents, extended family members, and siblings. ^2^Includes probands and affected sibling(s). Affected parents are not considered. ^3^Proportion of cases at least 18 years of age at the time of diagnosis (for schizophrenia) or time of DNA submission for the microarray genotyping (for ASD (*n* = 73)). ^4^Includes affected individuals for whom parents were not sampled or failed QC. No parents were sampled for ADHD. ^5^Affected individuals with one parent sampled or passed QC. ^6^Proband and both parents. ^7^Proband, one affected sibling and both parents. ^8^Families with three or more affected individuals (15 families with three, one family with four, and one with five affected children) for ASD. ^9^Includes 231 samples of extended family members or parents, including affected individuals. ^10^Of SCZ cases, 31 had intellectual disability

### Clinically relevant CNVs

Clinically relevant CNVs included five categories: aneuploidies, large CNVs ( > 3 Mb), CNVs consistent with known recurrent genomic disorders, those impacting genes previously established to be associated with NDDs, and all de novo CNVs (i.e., not found in either parent) (details in Table 2). We found 306 clinically relevant CNVs in 284 of 2,691 NDD cases (10.5%) (Tables 2, 3, Supplementary Table 1C). Of these CNVs, 115 found in 111/2,691 cases (4.3%) were “clinically significant” or “likely clinically significant” variants, as evaluated by expert clinical geneticists according to American College of Medical Genetics and Genomics guidelines.^26^ We did not find evidence for uniparental disomy. The clinically relevant autosomal deletions, and chromosome X deletions in females, were all single copy.

**Table 2:** Summary of cases carrying a CNV deemed relevant to neurodevelopmental disorders CNVs stratified by the disorder and variant type

Category	ASD (%)	ADHD (%)	OCD (%)	SCZ (%)	All cases (%)
A: aneuploidies	11 (0.6)	6 (1.4)	0	0	17 (0.63)
B: large CNVs (>3 Mb)^1^	16 (0.9)	4 (0.9)	0	3 (1.5)	23 (0.85)
C: genomic disorder loci^2^	80 (4.3)	18 (4.2)	4 (1.8)	13 (6.4)	115 (4.3)
D: de novo^3^	31 (6.9)	NA^5^	3 (1.8)	1 (NA)^6^	35 (5.6)
E: others^4^	89 (4.9)	14 (3.3)	8 (3.6)	6 (2.9)	117 (4.4)
Sum of unique samples across all categories^7^	209 (11.4)	40 (9.4)	13 (5.6)	22 (10.8)	284 (10.5)^8^

^1^This category included variants larger than 3 Mb but not aneuploidies or variants of known recurrent genomic disorder loci larger than 3 Mb, i.e., 15q11-13 duplication (Table 3, Supplementary Table 1C). ^2^Prevalence of known recurrent genomic syndromes in the general population is 0.8–1.0%. ^3^This category includes de novo variants from aneuploidies, large CNVs and genomic disorder loci. The rate of de novo CNVs in the general population is 0.9–1.4%^43^. ^4^This category included all other NDD relevant CNVs. ^5^Indicates that we did not sample parents of ADHD cases to establish inheritance pattern of variants. ^6^We sampled only 13 trios; most SCZ samples were unrelated. Sum of the counts in each column is higher in some cases than the total number of cases with relevant CNVs in the corresponding column, due to the fact that (i) some subjects might carry multiple relevant variants, and (ii) de novo category includes CNVs from aneuploidy, large CNVs, and the genomic disorder loci. ^7^Numbers of prioritized CNVs for ASD, ADHD, OCD, and SCZ were 223, 43, 17, and 23, respectively. ^8^Total number of prioritized CNVs across all cases was 306. The coordinates for clinically relevant CNVs are in Supplementary Table 1C

**Table 3:** Copy number variations of clinical significance among four neurodevelopmental disorders

Category	#^1^	Cytoband	Type	Size (kb)	Sex	Disorder	Class^2^	Origin
A: aneuploidies (*n* = 17)								
45,X	2	Xp22.33-q28	DEL	155,271	F	ASD, ADHD, SCZ^A^	CS	D, U
47,XX, + 21/47,XY, + 21	6	21p13-q22.3	DUP	48,130	F(2), M(4)	ASD	CS	D, U(5)
47,XXY	5	Xp22.33-q28	DUP	155,271	M	ASD(2), ADHD(3), SCZ^A^	CS	U
47,XYY	4	Yp11.32-q12	DUP	59,374	M	ASD(2), ADHD(2), SCZ^A^	CS	D(2), U(2)
B: large CNVs ( > 3 Mb; *n* = 23) excluding aneuploidies (category A) and CNVs associated with known recurrent genomic disorders (category C)								
46,XX,dup(2)(q23.3q24.1)	1	2q23.3-q24.1	DUP	3,071	F	ASD	VUS	U
46,XY,del(3)(p14.1p13)	1	3p14.1-p13	DEL	5,301	M	ASD	LCS	D
46,XY,dup(4)(q22.1q22.2)	1	4q22.1-q22.2	DUP	3,060	M	ASD	VUS	U
46,XY,del(4)(q32.3)	2*	4q32.3	DEL	3,209	M	ASD	VUS	U
46,XX,dup(4)(q34.3q35.1)	1	4q34.3-q35.1	DUP	3,886	F	SCZ, SCZ^A^	VUS	U
46,XY,dup(6)(q15q16.1)	1	6q15-q16.1	DUP	4,739	M	ASD	LCS	M
46,XX,dup,(6)(p26q27)	1	6p26-q27	DUP	7,302	F	SCZ, SCZ^A^	LCS	U
46,XY,del(7)(q31.1q31.31)	1	7q31.1-q31.31	DEL	11,051	M	ASD, SCZ^A^	CS	D
46,XY,del(8)(p23.3p23.1)	1	8p23.3-p23.1	DEL	6,841	M	ASD, SCZ^A^	CS	U
46,XY,del(8)(p23.3p22)	1	8p23.3-p22	DEL	14,917	M	ADHD	CS	U^3^
46,XY,del(8)(q12.3q13.2)	1	8q12.3-q13.2	DEL	3,278	M	ASD	LCS	U
46,XY,del(8)(q24.21q24.22)	1	8q24.21-q24.22	DEL	4,945	M	ADHD	LCS	U
46,XY,del(9)(p22.1p21.2)	1	9p22.1-p21.2	DEL	8,005	M	ASD	CS	U
46,XY,del(13)(q14.13q14.3)	1	13q14.13-q14.3	DEL	5,350	M	SCZ	LCS	U
46,XY,dup(15)(q25.3q26.1)	1	15q25.3-q26.1	DUP	6,519	M	ASD	LCS	U
46,XY,dup(16)(q11.2q21)	1	16q11.2-q21	DUP	15,626	M	ASD	CS	U
46,XY,dup(16)(q11.2q13)	1	16q11.2-q13	DUP	10,873	M	ASD	CS	D
46,XX,dup(17)(q11.1q12)	1	17q11.1-q12	DUP	6,145	F	ASD	CS	U
46,XY,del(18)(p11.32p11.21)	1	18p11.32-p11.21	DEL	15,264	M	ASD	CS	U
46,XY,del(18)(q22.1q23)	1	18q22.1-q23	DEL	13,469	M	ASD	CS	U
46,XX,dup(22)(q11.21q12.3)	1	22q11.21-q12.3	DUP	12,518	F	ADHD	CS	U
46,XY,del(Y)(p11.2)	1	Yp11.2	DEL	3,140	M	ADHD	VUS	U
C: CNVs associated with known recurrent genomic disorders (*n* = 115)								
1q21.1 Proximal Deletion (TAR Syndrome)	3	1q21.1	DEL	410–513	M	ASD	VUS	M(2), U
1q21.1 Distal Duplication	4	1q21.1-q21.2	DUP	1,817–2,034	F(2), M(2)	ADHD, ASD(3), SZC^A^(2)	CS	M, U(3)
2q37.3 Deletion	1	2q37.3	DEL	5,275	F	ADHD	CS	U
3q29 Deletion	1	3q29	DEL	2,603	M	ASD	CS	U
4p16.3 Duplication	2^ǂ^	4p16.3	DUP	846-849	M	ASD	VUS	P
5p Deletion (Cri-du-chat)	1	5p15.33-p15.2	DEL	10,191	F	SCZ, SZC^A^(2)	CS	U
7q11.23 Deletion (WBS; includes *ELN*)	1	7q11.23	DEL	1,429	M	ASD	CS	D
7q11.23 Duplication	1	7q11.23	DUP	716	M	ASD	VUS	U
9q34.3 Duplication	1	9q34.3	DUP	2,064	M	ASD	LCS	U
10q11.22-q11.23 Duplication	2*	10q11.22-q11.23	DUP	2,650–2,661	F	ASD, SCZ^A^	VUS	D
15q11-q13 Duplication	6	15q11.2-q13.1	DUP	4,918–6,158	F(2), M(4)	ADHD, ASD(3), OCD, SCZ, SCZ^A^(6)	CS	U
15q11.2 Deletion (BP1-BP2)	16	15q11.2	DEL	312–521	F, M(15)	ADHD(3), ASD(12), OCD	VUS	M(4), U(12)
15q11.2 Duplication (BP1-BP2)	15	15q11.2	DUP	311–850	F(7), M(8)	ADHD(3), ASD(12)	LB	M(2), P(2), U(11)
15q13.3 Deletion	4	15q13.2-q13.3	DEL	1,533–2,072	F(2), M(2)	ASD, SCZ^A^	CS	P, U(3)
15q25 Distal Deletion	1	15q25.2-q25.3	DEL	959	F	ASD	VUS	U
Rubinstein-Taybi Syndrome	1	16p13.3	DUP	204	M	ASD	VUS	U
16p13.11 Deletion	3	16p13.11-p12.3	DEL	1,496–3,352	F, M(2)	ADHD, ASD(2)	CS, VUS(2)	D, M, U
16p13.11 Duplication	17	16p13.11-p12.3	DUP	783–2,950	F(3), M(14)	ADHD, ASD(12), OCD, SCZ(3), SCZ^A^	VUS	M(3), P(3), U(11)
16p12.1 Duplication	3	16p12.2	DUP	670–680	F, M(2)	ASD	VUS	M(2), U
16p12.1 Deletion	3	16p12.2	DEL	613–655	M	ADHD(2), ASD	VUS	U
16p11.2 Distal Duplication	3	16p11.2	DUP	273–362	F, M(2)	ADHD, ASD, SCZ	CS	U
16p11.2 Distal Deletion	2	16p11.2	DEL	227–243	F, M	ASD	CS	D, U
16p11.2 Proximal Duplication	1	16p11.2	DUP	625	M	ADHD, ADHD^A^, SCZ^A^(4)	CS	U
16p11.2 Proximal Deletion	4	16p11.2	DEL	598–746	F, M(3)	ADHD, ASD(2), SCZ, SCZ^A^	CS	U
17p12 Deletion	1	17p12	DEL	1,404	F	OCD	CS	M
17p12 Duplication	2	17q12	DUP	1,858–1,970	M	ADHD, ASD	CS	U
22q11.21 Deletion	7	22q11.21	DEL	1,396–3,154	F(3), M(4)	ASD, SCZ(6), SCZ^A^(6)	CS	U
22q11.21 Duplication	7	22q11.21	DUP	2,546–3,271	M	ASD	CS	D, P, U(5)
22q11.2 Distal Duplication (LCR22-F to LCR22-H)	1	22q11.22-q11.23	DUP	2,062	F	ASD	LCS	U
22q13 Deletion	1	22q13.33	DEL	507	F	ASD	CS	U
Xp22.3 Deletion	1	Xp22.3	DEL	1,681	M	ADHD	CS	U
D**:** de novo (not in A to C; *n* = 23)								
1q21.3 Deletion (*PSMD4* + 3 genes)	1	1q21.3	DEL	98	M	ASD	VUS	D
2q23.1 Deletion (*MBD5* *+* *ORC4*)	1	2q23.1	DEL	251	M	ASD	CS	D
2q24.1 Duplication (*NR4A2*, *GPD2*)	1	2q24.1	DUP	1,090	M	ASD	VUS	D
2q32.1-q32.2 Deletion (*GULP1*)	1	2q32.1-q32.2	DEL	328	F	ASD	VUS	D
4p16.3 Deletion (*ADRA2C*)	1	4p16.3	DEL	165	M	OCD	VUS	D
7p22.1 Deletion (*AP5Z1, FOXK1*)	1	7p22.1	DEL	44	M	ASD	VUS	D
7p22.1 Deletion (*FBXL18* *+* *TNRC18*)	1	7p22.1	DEL	130	M	ASD	VUS	D
7q11.22 Deletion (*AUTS2*)	1	7q11.22	DEL	428	M	ASD	LCS	D
7q36.3 Duplication (*EN2*, *RBM33*, *CNPY1*)	1	7q36.3	DUP	341	F	ASD	VUS	D
8p23.3 Duplication (*DLGAP2*)	1	8p23.3	DUP	829	M	ASD, SCZ^A^	LB	D
10q11.21-q11.22 Duplication (*ZFAND4*, *MARCH8*, *WASHC2C*)	1	10q11.21-q11.22	DUP	239	F	OCD	VUS	D
16p13.2 Duplication (*USP7* + 3 genes)	1	16p13.2	DUP	326	F	ASD	VUS	D
16q23.3-q24.1 Deletion (*ATP2C2* + 22 genes)	1	16q23.3-q24.1	DEL	1,900	M	ASD	VUS	D
17p13.3 Deletion (*INPP5K, PITPNA*, *SLC43A2*)	1	17p13.3	DEL	102	M	ASD	VUS	D
17q25.3 Duplication (*ACTG1* + 37 genes)	1	17q25.3	DUP	859	M	ASD	VUS	D
17q25.3 Deletion (*CSNK1D*, *SLC16A3*)	1	17q25.3	DEL	63	M	ASD	VUS	D
18p11.32 Duplication (*COLEC12* + 7 genes)	1	18p11.32	DUP	547	F	ASD	VUS	D
19q13.33 Duplication (*GRIN2D*, *KCNC3*, *PNKP* + 107 genes)	1	19q13.33	DUP	2,645	F	ASD	LCS	D
21q22.3 Duplication (*PDE9A* *+* 4 genes)	2^	21q22.3	DUP	42–284	M	ASD	VUS	D
22q11.23 Duplication (*UPB1*)	1	22q11.23	DUP	21	F	SCZ	VUS	D
Xp22.31 Duplication (*ANOS1*, *VCX3B*)	1	Xp22.31	DUP	305	F	OCD	VUS	D
Xp11.22 Deletion (*SMC1A)*	1	Xp11.22	DEL	24	F	ASD	CS	D
E: CNVs not in categories A-D (*n* = 127)								
1p36.33-p36.32 Deletion (*SKI* + 61 genes)	1	1p36.33-1p36.32	DEL	1,779	M	ASD	CS	U
1q21.1 Duplication (*HFE2* + 19 genes)^3^	1	1q21.1	DUP	871	F	ASD, SCZ^A^	VUS	U
*POGZ, PSMB4, SELENBP1*	1	1q21.3	DUP	73	M	ASD	B	U
*DISC1*	1	1q42.2	DUP	26	M	ASD	VUS	U
*NRXN1*	10	2p16.3	DEL	35–658	F, M(9)	ADHD, ASD(6), SCZ(3), SCZ^A^(2)	CS	P(4), U(6)
*DPP10*	1	2q14.1	DEL	472	F	ASD	VUS	P
*CNTNAP5*	1	2q14.3	DUP	1,043	M	ASD	VUS	U
*MBD5*	1	2q23.1	DUP	315	M	SCZ	LCS	U
*MBD5*	4	2q23.1	DEL	57-208	M	ASD	LCS	M(2), P, U
*CNTN4* + *CNTN6*	2	3p26.3-3p26.2	DUP	1,734–2,049	M	ASD, OCD	VUS	P, U
*CNTN4*	1	3p26.3	DUP	54	F	ASD	VUS	M
*CNTN6*	1	3p26.3	DEL	131	M	ASD	B	U
*SUMF1*, *ITPR1*	1	3p26.1	DEL	277	M	SCZ, SCZ^A^	CS	M
*GRM7*	1	3p26.1	DUP	1,738	M	ASD	VUS	U
*NLGN1*	1	3q26.31	DEL	142	M	OCD	VUS	M
*PAK2*	2	3q29	DEL	31-37	M	ASD	VUS	U
*DLG1* + *BDH1*	1	3q29	DUP	341	M	ASD	LB	U
*ANKRD17* + *COX18*	1	4q13.3	DUP	236	M	ASD	LB	M
*GRID2*	2	4q22.2	DEL	85–256	M	ADHD, ASD	VUS	P, U
*ANK2, PITX2* + 9 genes	1	4q25	DUP	2,841	F	ASD, SCZ^A^	VUS	U
*CTNND2*	2^ǂ^	5p15.2	DUP	81	M	ASD	VUS	M
*CTNND2*	1	5p15.2	DEL	52	F	ASD	VUS	U
*MEF2C, TMEM161B*	2	5q14.3	DUP	109–2,043	F, M	ASD	VUS	U
*MEF2C*	1	5q14.3	DEL	272	F	ASD	VUS	U
*PTPRK*	2^ǂ^	6q22.33	DEL	130-135	M	ASD	VUS	U
*LAMA2*, *ARHGAP18*	1	6q22.33	DUP	1,064	M	ASD	VUS	P
*AUTS2*	1	7q11.22	DUP	344	F	ASD	VUS	M
*ELN* + 4 genes	1	7q11.23	DUP	320	M	ASD	LCS	U
*GRM8*	1	7q31.33	DEL	73	M	ASD	VUS	U
*CNTNAP2*	1	7q35	DEL	135	M	ASD	LB	M
*KMT2C*	1	7q36.1	DUP	440	F	ASD	LB	U
*DPP6*, *PAXIP1*, *HTR5A*	3	7q36.2	DUP	123–1,800	M	ADHD, ASD(2)	VUS	M, U(2)
*DPP6*	3	7q36.2	DEL	52–396	M	ADHD, ASD, OCD, SCZ^A^	VUS	M, P, U
*PTPRN2*, *ESYT2*, *NCAPG2*	3	7q36.3	DUP	50–430	M	ADHD, ADHD^A^, ASD, OCD	LB	M, P, U
*DLGAP2*, *CLN8, ARHGEF10*	3	8p23.3	DUP	317–358	M	ASD	VUS	P
*DLGAP2*	2	8p23.3	DEL	58–191	M	ADHD, ASD	VUS	U
*MCPH1*	2	8p23.2-8p23.1	DUP	181–272	M	ADHD, OCD	LB	M, U
*PTPRD*	1	9p24.1	DEL	81	M	ASD	VUS	U
*ASTN2*, *TRIM32, PAPPA*	3	9q33.1	DEL	25–523	F, M(2)	ADHD^A^(2), ASD	VUS	U
*PCDH15*	2	10q21.1	DEL	58–291	F, M	ASD	VUS	U
*CTNNA3*	7	10q21.3	DEL	53–306	F(2), M(5)	ASD	B	M(3), P, U(3)
10q24.32 Duplication (*POLL*, *BTRC*, *DPCD*)	1	10q24.32	DUP	226	M	ASD	LCS	P
*DRD4* *+* 8 genes	1	11p15.5	DUP	154	M	ADHD	VUS	U
*PAX6, ELP4*	1	11p13	DUP	393	M	ADHD	VUS	U
*SHANK2*	1	11q13.4	DEL	132	M	ASD	LCS	M
*DLG2*	1	11q14.1	DEL	210	F	ASD	VUS	U
*CNTN5*	1	11q22.1	DUP	25	M	ASD	VUS	U
*KMT2A* *+* 6 genes	1	11q23.3	DUP	265	F	ASD	VUS	M
*CACNA1C*	1	12p13.33	DUP	56	M	ASD	VUS	P
*PCDH9*	1	13q21.32	DUP	1,006	M	ASD	VUS	M
*CHD8*	1	14q11.2	DUP	23	M	ASD	VUS	P
*NRXN3*	3^±^	14q31.1	DEL	224–254	F, M(2)	ASD	LCS	P(2), U
15q13.1-q13.2 Duplication (*APBA2* + 4 genes)	1	15q13.1-q13.2	DUP	1,376	M	ASD	VUS	M
*CHRNA7*	1	15q13.3	DEL	441	F	ASD	LCS	P
*RBFOX1*	1	16p13.3	DEL	103	F	ASD, SCZ^A^(2)	VUS	U
*RBFOX1*	2	16p13.3	DUP	42–373	M	ASD	VUS	U
16p11.2 Duplication (*ATP2A1* + 20 genes)	1	16p11.2	DUP	962	F	ASD	VUS	U
*ATP2C2* *+* 11 genes	1	16q24.1	DUP	1,061	F	ASD	VUS	U
*ATP2C2*, *TLDC1*	3	16q24.1	DEL	23–126	M	ADHD	LB	U
*ANKRD11* + 5 genes	1	16q24.3	DUP	250	M	ASD	VUS	U
*NF1* + 14 genes	3	17q11.2	DUP	83–1,394	F, M(2)	ADHD, ASD, SCZ	LCS, VUS(2)	P, U(2)
*DLGAP1*	1	18p11.31	DUP	62	M	OCD	VUS	P
*CDH7, CDH19*	1	18p22.1	DEL	2,126	F	OCD	VUS	M
*MACROD2*	1	20p12.1	DUP	22	M	ASD	VUS	U
*MACROD2*	2	20p12.1	DEL	72–237	M	ADHD^A^(2), ASD, OCD, SCZ^A^	VUS	U
*PTPRT* + 6 genes	1	20q12-q13.12	DUP	836	M	ASD, SCZ^A^	VUS	U
*PTPRT*	1	20q12	DEL	190	F	OCD	VUS	U
22q11.21 Deletion (*SNAP29*, *LZTR1* + 12 genes)	1	22q11.21	DEL	749	M	ADHD	LCS	U
*CACNA1I*	1	22q13.1	DUP	116	M	ASD	VUS	U
*ARSE* + 7 genes	2	Xp22.33-Xp22.32	DUP^4^	2,262	M	ASD	VUS	P
*ANOS1* + 4 genes	1	Xp22.31	DUP	1,247	F	OCD	VUS	U
*PTCHD1-AS* + 5 genes	5	Xp22.11	DEL	81–1,169	M	ADHD^A^, ASD	VUS	M(4), U
*IL1RAPL1*	1	Xp21.3-Xp21.2	DUP	546	M	ASD	LCS	M
*DMD*, *TAB3, FTHL17*	2	Xp21.2-Xp21.1	DUP	252–792	M	ASD	LCS,VUS	M
*RAB38B* + 8 genes	1	Xq28	DUP	286	F	ADHD	VUS	U

*WBS* Williams-Beuren Syndrome, *F* female, *M* male, *DEL* deletion, *DUP* duplication, *ASD* autism spectrum disorder, *ADHD* attention deficit hyperactivity disorder, *SCZ* schizophrenia, *OCD* obsessive compulsive disorder, *VUS* variants of unknown significance, *B* benign, *LB* likely benign, *CS* clinically significant, *LCS* likely clinically significant, *M* maternally inherited, *P* paternally inherited, *U* unknown inheritance, *D* de novo. ^1^Number of subjects carrying CNVs in the indicated cytoband. ^2^Clinical impact of variants was evaluated according to accepted clinical guidelines. ^3^Mosaic CNV. ^4^These two duplications are in two brothers. qPCR showed that the father was also carrying these duplications on chromosome X, which implies that there might be translocation between X and an unknown autosome, which then was transmitted to these two boys. We did not attempt identify the location of this duplication on autosomes. ^A^Indicates the presence of a similar CNV detected in a subject published previously as part of two large-scale schizophrenia CNV studies (Costain et al. ^24^ and Lowther et al.^11^) and one ADHD study (Lionel et al.^10^). Sex, size of CNVs, inheritance, and the clinical classifications of these CNVs were not provided

Only genes whose coding sequences are impacted by CNVs are shown. However, CNVs impacting exons (UTRs) in *MBD5* and *PTCHD*-*AS* were indicated

*Monozygotic pair of twins from two different families; ^ǂ^Siblings; ^two duplications from one case. ^±^two of these deletions are paternally inherited in siblings diagnosed with ASD (one male, one female)

The coordinates for clinically relevant CNVs are in Supplementary Table 1C

**Fig. 1:**
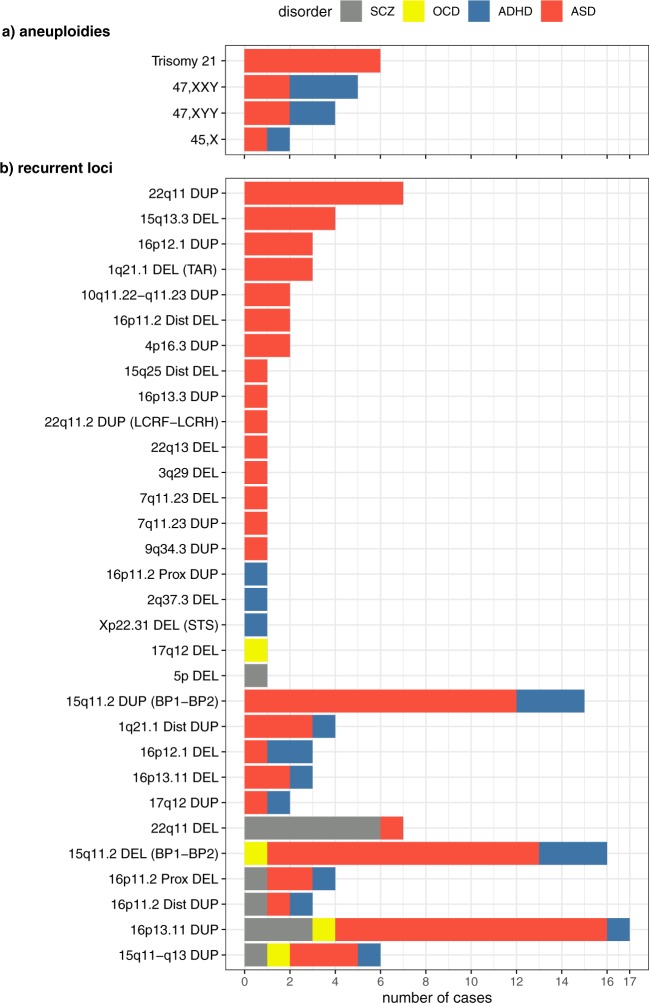
Distribution of **a**) aneuploidies and **b**) known recurrent genomic disorder CNVs found in cases diagnosed with autism spectrum disorder (ASD), attention deficit hyperactivity disorder (ADHD), schizophrenia (SCZ), or obsessive compulsive disorder (OCD). Details of the copy number variants, sex, and variant sizes are in Table 1, Supplementary Table 1A. *DEL* deletion, *DUP* duplication, *TAR* Thrombocytopenia-Absent Radius syndrome locus, *STS* includes STS, *BP* breakpoint, *LCR* low-copy repeat, *Prox* proximal, *Dist* distal

The first category included aneuploidies: trisomy 21, 47,XXY, 47,XYY, and 45,X, found in 17/2,691 cases (0.63%), only among ADHD or ASD cases (category A in Tables 2, 3; Fig. 1). The second CNV category contained variants larger than 3 Mb, but excluding those associated with recurrent genomic disorders and aneuploidies (category B in Tables 2, 3). We found these large variants in 23/2,691 NDD cases (0.85%), with none among the OCD cases. The third category was the variants associated with known recurrent genomic disorders, found in 115/2,691 cases (4.3%; category C in Tables 2, 3; Fig. 1). The most frequent were 16p13.11 duplications (17 cases), 15q11.2 deletions (breakpoint (BP1-BP2) (16 cases), and 15q11.2 duplications (BP1-BP2) (15 cases). Three distal duplications of 16p11.2 were found in ADHD, ASD, and SCZ cases. We found 15q11-q13 duplications in six cases diagnosed with ADHD, ASD, OCD, or SCZ. The high prevalence of 15q11.2 duplications (BP1-BP2) and 16p13.11 duplications likely reflects the relatively mild expression and reduced penetrance of these genotypes.

Fourth, we identified de novo CNVs impacting genes in 35 cases: 31/448 (6.9%) for ASD and 3/167 (1.8%) for OCD (category D in Tables 2, 3). Of 13 SCZ trios with data, we identified one de novo genic CNV. Parents of ADHD cases were not analyzed, thus no data on de novo CNVs were available for this disorder.

The last class of variants included inherited clinically relevant CNVs that did not belong to any of the first three categories. These included CNVs impacting genes previously implicated for NDDs but either inherited or with unknown inheritance. We found these variants in 117/2,691 cases (4.4%) (category E in Tables 2, 3).

### Cases with more than one clinically relevant CNV

We found more than one clinically relevant CNV in 20/2,691 (0.74%) of NDD cases: 12/1,838 (0.65%) of ASD cases, 4/427 (0.95%) of ADHD cases; 3/222 (1.35%) of those with OCD, and 1/204 (0.5%) of individuals with SCZ.

In ASD, there were eleven cases with two CNVs and one case with three relevant variants. The latter, case 4-0040-003, had a maternally inherited 53 kb deletion impacting *CTNNA3*, a maternally inherited 54 kb duplication impacting *CNTN4*, and a de novo 547 kb duplication impacting *YES1, ADCYAP1*, and six other genes. Examples with two clinically relevant CNVs each included: (i) a male (7-0293-003) with a 611 kb deletion of 16p11.2 and a 513 kb deletion of 15q11.2, of unknown inheritance; (ii) a female (2-1525-003) with a maternally inherited 1.5 Mb deletion consistent with the 16p13.11 recurrent microdeletion (neurocognitive disorder susceptibility locus), and a de novo 326 kb duplication impacting *USP7*, *PMM2*, *C16orf72*, and *CARHSP1*; and (iii) a male (2-0305-004) with a maternally inherited 432 kb deletion in the 1q21.1 locus associated with thrombocytopenia-absent radius syndrome and a de novo 1.09 Mb duplication impacting *GPD2* and *NR4A2*.

The four cases with ADHD with more than one CNV were all male; each had two clinically relevant CNVs, all of unknown inheritance. Examples included: (i) 213050 with Klinefelter syndrome (47,XXY) and an autosomal 181 kb duplication affecting *MCPH1*; (ii) 206760 with a 191 kb deletion impacting *DLGAP2* and a 154 kb duplication involving *DRD4* and eight more genes; and (iii) 235983S with Klinefelter syndrome (47,XXY) and an autosomal 123 kb duplication impacting *DPP6*.

Of the three OCD cases carrying more than one CNV, there was one male (OCD146-JS-1254_188613) with three clinically significant CNVs: a maternally inherited 143 kb deletion impacting *NLGN1*, a second maternally inherited 115 kb deletion impacting *DPP6*, and a paternally inherited 67 kb duplication impacting *PTPRN2*. Another male (OCD125-896993) had a de novo 165 kb deletion impacting *ADRA2C* and a paternally inherited 1.7 Mb duplication impacting *CNTN4* and *CNTN6*. A female (OCD109-1648) had a maternally inherited 1.4 Mb deletion of 17p12 and a 190 kb deletion impacting *PTPRT*. This case also had a de novo frameshift deletion in *LRCH2* (c.2190_2193del:p.C730fs; Supplementary Table 1C) identified using WES.

The only individual with SCZ having two clinically relevant CNVs (Supplementary Table 1C) was a female (222720) with a 10.2 Mb deletion of chromosome 5p congruent with the cri-du-chat syndrome region, and a 7.3 Mb duplication of 6q26-27. Karyotyping confirmed this to be the result of an unbalanced translocation.^11^

### Cases with clinically relevant CNVs identified by microarray also had SNVs or CNVs detected by WGS or WES

No WGS data were available for the ADHD and SCZ samples. However, we have previously published WGS data for 106/209 (50.7%) ASD cases with clinically relevant CNVs.^8,27,28^ Of these, 15 had a clinically relevant LoF mutation and one had a 4.5 kb deletion (Supplementary Table 1C). The latter case was a male (2-1086-004) with a maternally inherited 80.1 kb duplication impacting *CTNND2*, identified using array data. He also had paternally inherited 4.5 kb deletion in *ANO3*, known to be associated with autosomal dominant dystonia (OMIM: 610110). This deletion was missed by microarray for lack of probes in this region, due to its size (Supplementary Table 1C). Examples of LoF SNVs were: (i) a female autism case (7-0133-003) with a 2.5 Mb de novo duplication in 10q11.22-11.23 and a de novo nonsense mutation in *SOX5* (c.C313T:p.R105X). Mutations of *SOX5* cause autosomal dominant Lamb-Shaffer syndrome, characterized by global developmental delay and intellectual disability (OMIM:604975), and (ii) a male autism case (7-0123-003) with a 2.9 Mb duplication involving the 16p13.11 recurrent microduplication who also had a de novo splice-site variant impacting *SHANK3* (c.2223 + 1 G > A), which is a gene strongly associated with NDDs.^8^ Of 13 OCD cases with clinically relevant CNVs, three also had clinically relevant LoF mutations previously identified using WES (Supplementary Table 1C).^13^ One example is a male OCD case (OCD146-JS-1254_188613) with three clinically relevant CNVs: a maternally inherited 142 kb deletion in 3q26.31, a maternally inherited 115 kb deletion in 7q36.2, and a paternally inherited 67 kb duplication in 7q36.3. He also had three clinically relevant SNVs found by WES: (i) a maternally inherited frameshift deletion in *AFF2* (c.2976_2988del:p.992_996del), which is an X-linked recessive variant associated with mental retardation (OMIM:300806), (ii) a maternally inherited frameshift deletion in *DRD4* (c.233_245del:p.A78fs), an autosomal dominant variant associated with autonomic nervous system dysfunction and ADHD (OMIM:126452), and (iii) a maternally inherited frameshift in *MBD4* (c.939_940ins:p.E314fs), a gene involved in DNA methylation (OMIM:603574).

### Complex phenotypes

Because we had clinical information on NDD phenotypes beyond the primary diagnoses for some cases, we investigated the pleiotropy of CNVs shared among different NDDs (Supplementary Table 1C). We defined complex as having multiple different NDDs. Of ADHD cases with an NDD relevant CNV, a few were noteworthy and highlight the clinical pleiotropy associated with many of these variants. A male ascertained for ADHD (176004), who also had a learning disability but no ASD, carried a duplication of 16p11.2, which is known to be associated with ASD.^8^ We found deletions at this locus in a female with SCZ, a male with ASD, and another male with ADHD, but none in our OCD cases. A male diagnosed with ADHD (206773), who carried a duplication of chromosome X (Klinefelter syndrome), also had ASD, learning disability, language delay, general anxiety disorder, and enuresis, all known feature of Klinefelter syndrome.^29^ Male case 181220 with ADHD, with 15q11.2 duplication (BP1-BP2), also had ASD. Of OCD cases, a male (OCD75-SB-1213) with a paternally inherited 62 kb duplication of *DLGAP2* also had separation anxiety disorder, a Tourette disorder with tic, oppositional defiant disorder, and panic disorders and agoraphobia. Another male with OCD (OCD146-JS-1254_188613), ADHD (inattentive subtype), and a Tourette disorder with tic, had three different CNVs, impacting *NLGN1*, *DPP6*, and *PTPRN2*. Of SCZ cases, a male (153030) with a 1.6 Mb duplication of 16p13.11 also had a learning disorder but no ID (details in Supplementary Table 1C). A female (213684) with SCZ and a 549 kb deletion of *NRXN1* also had moderate intellectual disability. A male with SCZ (166808) with a 15q11-q13 duplication had mild intellectual disability.

### Cross-disorder gene discovery and genes in multiple cases in a single disorder

**Fig. 2:**
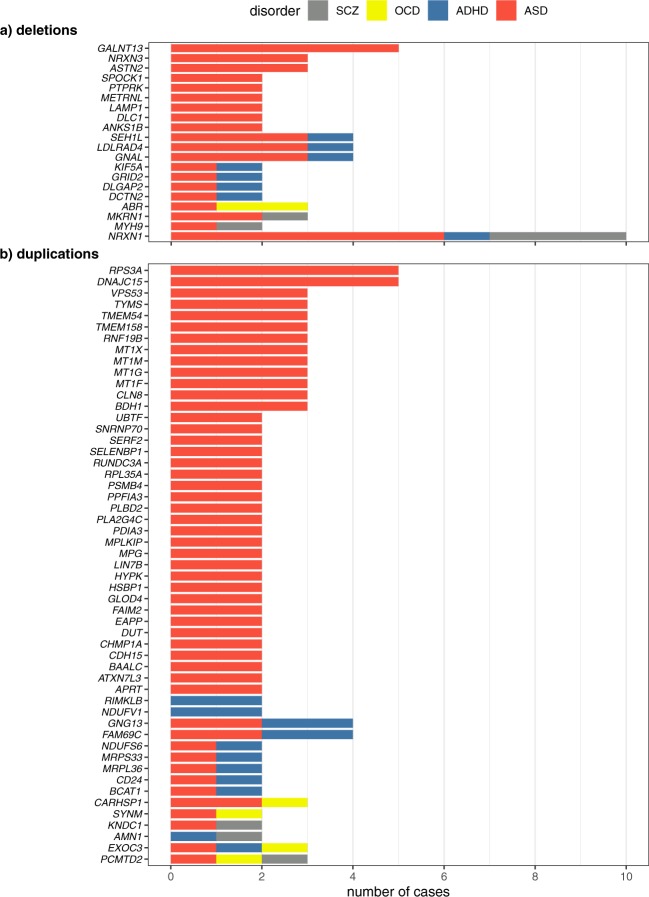
Genes impacted by rare CNVs in more than one case. **a**) brain-expressed and moderately constrained genes (pLI > 0.45) impacted by deletions in multiple cases, **b**) brain-expressed genes with duplication of their full-length transcript in more than one case

We searched for genes, excluding those from regions of known recurrent genomic disorders, that were affected in multiple cases by CNVs. We first restricted analysis to brain-expressed genes that are at least moderately constrained for LoF variants, (pLI > 0.45; Fig. 2; Supplementary Table 1D). We searched these for genes impacted by CNVs in at least two cases each, and found 20 genes impacted by deletions. Notably, *NRXN1* was impacted in 10 subjects (three SCZ, six ASD, and one ADHD); deletions of 18p11.21 impacting novel candidate genes (*GNAL, LDLRAD4*, and *SEH1L)* were in four cases (three ASD, one ADHD). Genes *MKRN1* and *MYH9* involved CNVs in ASD and SCZ cases. Eight genes – *NRXN1, GNAL, LDLRAD4, SEH1L, DLGAP2, DCTN2, GRID2*, and *KIF5A* – involved CNVs in ASD and ADHD cases. Only CNVs containing *ABR* were shared between OCD and ASD, and no gene-containing variants were shared between OCD and ADHD or OCD and SCZ.

Genes impacted by deletions in multiple ASD cases (only) were: *ASTN2*, *NRXN3*, *ANKS1B*, *GALNT13*, *DLC1*, *LAMP1*, *METRNL*, *PTPRK*, and *SPOCK1* (Fig. 2; Supplementary Table 1D). Although *ASTN2* deletions were previously reported in ADHD,^30^ in this study we made no such observations. We saw no genes impacted by deletions in multiple cases of ADHD, OCD, or SCZ, other than those in the known genomic syndromes (Fig. 2; Supplementary Table 1E).

We identified 53 brain-expressed genes impacted in multiple cases by duplications of the entire longest transcript (Fig. 2; Supplementary Table 1E). Examples included *DNAJC15*, *GNG13*, *CARHSP1*, *PCMTD2*, *RPS3A*, and *TMEM158* (Supplementary Table 1E). Most whole-gene duplications were from ASD cases, probably due to the latter’s disproportionate representation. Examples of genes with such variants in multiple disorders were: *PCMTD2* in ASD, OCD and SCZ; *KNDC1* in ASD and SCZ; *CARHSP1*, *PCMTD2*, *SYNM*, and *EXOC3* in both ASD and OCD; and *GNG13*, *MRPS33*, *RPS3A*, *FAM69C*, and *CD24* in ASD and ADHD.

We found 38 genes duplicated in multiple ASD cases (only). Examples included *CDH15*, *UBTF*, *DUT*, *HYPK*, *ATXN7L3*, and *GLOD4*. Duplication of *NDUFV1* and *RIMKLB* were each observed in two ADHD cases (Fig. 2; Supplementary Table 1E). We found no repeated full gene duplications in OCD or SCZ cases in this collection.

### Increased burden of rare CNVs impacting brain-expressed protein coding genes and brain-expressed long non-coding RNA (lncRNA)

We sought rare CNVs impacting exons of lncRNAs and found these in 1,130/2,691 cases (42%). Restricting to brain-expressed lncRNAs, only 234/2,691 cases (8.7%) carried such rare CNVs. We tested for the extent to which the protein coding genes and lncRNAs were impacted by rare CNVs in cases compared with parents of cases. We found a nearly significant excess in cases over controls of deletions in protein-coding genes (*p* = 0.08; false discovery rate (FDR) = 0.21), but not for lncRNAs (*p* > 0.1). We found no global excess burden of duplications for protein-coding genes and lncRNAs (*p* > 0.1). However, when focused on brain-expressed elements, we observed a modest increase of rare deletions impacting both protein-coding genes (*p* = 0.03; FDR = 0.19) and lncRNAs (*p* = 0.06; FDR = 0.21). We then performed a multivariate analysis to test whether the burden signal was from protein-coding, lncRNAs, or both. This analysis showed a statistically significant signal (*p* = 0.02) for deletions impacting protein-coding genes, suggesting an overlap between the protein-coding and lncRNA burden signal.

Given the increasing association of lncRNAs in disease, we highlight two example of such genes identified in multiple unrelated individuals. (i) *AK127244*: three subjects with ASD (1-0045-004, 7-0103-003, and 1-0629-003) harbored 2p16.3 deletions that directly disrupted the exonic sequence *AK127244* (*LOC730100*). This is a 1.38 Mb non-coding RNA of unknown function adjacent to *NRXN1* and transcribed in the opposite direction. Rare, inherited deletions intragenic to *AK127244* have been identified in five individuals with ASD. Such deletions have been proposed as candidate factors for a broad range of neuropsychiatric disorders including SCZ and affective disorder.^16,31,32^ We identified seven additional subjects here, five with ASD and two with SCZ, with coding deletions of *NRXN1* that extend and disrupt the transcription start site and exonic sequence of *AK127244*. (ii) *PTCHD1-AS*: we found five males with ASD and deletions impacting exons of this gene (Table 3, Supplementary Table 1C). Disruption of *PTCHD1-AS* has been linked to ASD.^33,34^

### Increased burden of CNVs impacting NDD genes in cases carrying CNVs that impact genomic instability genes and fragile sites

We hypothesized that CNVs affecting genes involved in genome stability might lead to a higher incidence of additional variants. These subsequent variants could then add to the phenotypic complexity, by impacting genes involved in the development and functions of the nervous system. We therefore tested if individuals carrying a CNV that impacts “genomic instability genes” (GIG-CNV) have a higher burden of rare CNVs (measured as the number and the cumulative length of rare CNVs per individual) than do individuals not carrying such CNVs.^35^ We compiled a set of 958 protein coding “genomic instability genes” from the AmiGO database.^36^

The “genomic instability genes” were not disproportionately impacted by rare CNVs in cases compared with controls (parents or unaffected individuals for this analysis) (*p* > 0.1). In individuals who had a GIG-CNV, we found an increase in mean number of CNVs (3.3 vs 2.1; *p* = 2.11 × 10^-8^) and cumulative length of rare CNVs (4.4 Mb vs 315 kb; *p* = 2.11 × 10^-8^) compared to individuals without a GIG-CNV. We observed a similar trend excluding CNVs impacting the “genomic instability genes” from the burden analysis (mean number of rare CNVs: 2.8 vs 2.1; *p* = 0.003; cumulative length of rare CNVs: 564 kb vs 315 kb; *p* = 0.024). This difference was even higher when considering only cases (i.e. not controls)(mean number of rare CNVs: 2.9 vs 2.1; *p* = 0.013; cumulative length of rare CNVs: 686 kb vs 358 kb; *p* = 0.051). We also found a 2.77-fold increase in the number of cases with rare CNVs impacting NDD-associated genes (NDD-CNV)(*n* = 1,160; Supplementary Information) and a GIG-CNV (Fischer’s exact test, *p* = 4.7 × 10^−05^, odds ratio: 2.77[CI:1.66–4.54]), compared with cases with NDD-CNVs but without a GIG-CNV. We then excluded individuals with aneuploidy or a CNV impacting both “genomic instability genes” and NDD genes. We still observed the excess number of cases with NDD-CNVs and GIG-CNVs over those with NDD-CNVs only (odds ratio of 2.52[CI:1.50-4.16] (Fisher’s exact test, *p* = 2.7 × 10^-4^)).

We then tested whether there was an increase CNV burden among cases whose parents had a GIG-CNV. Such CNVs could have been generated de novo anywhere previously in the pedigree and had not necessarily arisen de novo in the affected individual. We excluded the cases carrying a de novo GIG-CNV that was not found in the parents. We found a higher average number of rare CNVs in cases whose parents had GIG-CNVs compared to cases whose parents did not (2.66 vs 2.10; *p* = 0.02). We observed a similar trend for this global burden when excluding the GIG-CNVs (2.49 vs 2.10; *p* = 0.08). However, we did not observe an increased global burden in the cumulative length of rare CNVs.

We then further investigated cases with de novo CNVs for whom WGS data were available (*n* = 16, all from the ASD cohort). We found no over-representation (*p* > 0.1) of cases with de novo CNVs from families in which at least one parent carried a LoF variant impacting a “genomic instability gene” (*n* = 10) compared to other families (*n* = 6). Again, this was a small sample size. There were notable examples of cases with de novo CNVs whose parents had LoF variant(s) on “genomic instability genes”. (i) Case 1-0627-007 had a paternally inherited frameshift deletion impacting *PALB2* (c.509_510del:p.R170fs). He also had a 1.9 Mb de novo deletion in 16q23.3-q241 (Table 3, Supplementary Table 1C). (ii) Case 2-1525-003 had a stop-gain mutation on *PALB2* (c.G2712A:p.W904X) and a 326 kb de novo duplication in 16p13.2. *PALB2* plays a role in homologous recombination and checkpoint response.^37^ (iii) Case 1-0181-004 had a paternally inherited variant in *EXO1* (c.G2482T:p.E828X) and a 5.3 Mb de novo deletion in 3p14.1-p13. *EXO1* functions in DNA replication, repair, and recombination (OMIM:606063). (iv) The father of ASD case 2-1693-003 had a *RAD1* variant (c.168_172del:p.A56fs) - a gene required for DNA replication and repair (OMIM: 603153). She carried a de novo 24 kb deletion at Xp11.22.

We also studied the extent of de novo CNVs overlapping genomic fragile sites. Of 33 de novo CNVs (excluding aneuploidies), 25 (75.6%) overlapped fragile site regions (Supplementary Table 1F). In addition, eleven of the de novo CNVs overlapped long genes (>300 kb), a feature associated with fragile sites and neuronal genes.^38^ Notably, five of these genes – *MBD5*, *FAM19A1*, *FOXP2*, *AUTS2*, and *DLGAP2* – are involved in neuron formation and differentiation.

## Discussion

We generated a bioresource to investigate the contribution of rare CNVs to the etiology of four NDDs – ASD, ADHD, OCD, and SCZ – among 2,691 diagnosed cases. We found that 10.5% of these cases carried CNVs with potential clinical relevance to NDDs. Of all cases, 4.1% carried CNVs that were formally classified as clinically significant or likely clinically significant, when evaluated according to ACMG guidelines.^26^ We also found variant genes/regions that were shared across some or all of the NDDs. Evidence included recurrent or non-recurrent CNVs impacting the same genes in cases with different NDDs, and in patients diagnosed with multiple comorbid NDDs.

Of the four NDDs, ASD had the highest proportion of cases with a clinically relevant CNV (11.4%). OCD cases had the lowest proportion with identified CNVs (5.6%). Deletions of 22q11.21 were found in 6/204 SCZ cases (2.9%) - three with mild intellectual disability - contributing to the relatively high proportion of SCZ cases deemed to have a clinically relevant CNV (10.8%). 22q11.2 deletions are expected to be identified in about one in every 100-200 individuals with SCZ and about one in 10 with dual diagnosis of SCZ and ID.^11^ The enrichment of the SCZ cohort studied for ID likely contributed more to the prevalence observed of 22q11.2 deletions. Of ADHD cases, 9.4% carried clinically relevant CNVs, which is slightly higher than 8.9% previously reported using a different microarray (Affymetrix SNP 6.0).^10^ We also found multiple clinically relevant CNVs in 20/2,691 (0.74%) of NDD cases.

We found 17 aneuploidies (45,X; 47,XXY and 47,XYY) in cases diagnosed with either ASD or ADHD (Table 2). The prevalence of Turner syndrome (45,X) (*n* = 2) was 1/1,300 among our cases, which is similar to previous reports.^39^ One had ADHD and the other ASD, similar to other reports.^39^ Cases with 47,XXY (*n* = 5) or 47,XYY (*n* = 4) had diagnoses of either ASD or ADHD, similar to previous reports.^29^ We found trisomy 21 (*n* = 6) only among ASD patients.^40^ Large CNVs other than aneuploidies were found in 23/2,691 (0.85%) cases, mainly in gene-rich regions of the genome. Although we did not find aneuploidies in SCZ cases, they have been reported in association with this phenotype previously.^11,24^

We found CNVs associated with known recurrent genomic disorders in 4.3% of cases (Table 2). This signified an increase of this type of CNVs among NDDs compared with that from a community population (1.1% (52/4,817); unpublished data), but similar to that of subjects with neurocognitive deficits in the UK Biobank (3.8%).^41^

Known recurrent genomic disorders were distributed differently among the four NDDs (Table 2; Fig. 1). We observed ASD in 80/115 (70%) of subjects with known recurrent genomic disorders, e.g., 7q11.23 deletion, 16p11.2 distal deletion, and 22q11.21 duplications. The 5p deletion was unique to SCZ. Deletions of 2q37.3, 16p11.2 proximal duplications, and Xp22.3 deletions were found only in ADHD, whereas 17q12 deletion was only found in OCD. Duplications of 15q11-q13, deletions of 15q11.2 (BP1-BP2), and 16p13.11 duplications were observed among cases of all four disorders (Fig. 1). Proximal duplications of 16p11.2 are found in up to 1% of individuals with SCZ.^11,24,42^

When parents were available to determine origin, we found 5.6% of this subset of cases to have a de novo CNV (Table 2). The highest de novo rate was for ASD (6.9%), consistent with previous reports from 4.7 to 7.1%.^43^ For OCD, 1.8% had de novo CNVs, which is higher than the rate found in the general population (0.9–1.4%),^43^ but lower than 2.3% for OCD previously reported from a larger sample size.^13^

We observed deletions and duplications, other than those associated with known recurrent genomic disorders, in the same genes in different NDDs, including some in multiple cases (Fig. 2; Table 2, Supplementary Table 1D). Deletions impacted *NRXN1* (in 9 males, 1 female) among ASD, ADHD, and SCZ cases (Fig. 2). Similarly, we found deletions impacting *GNAL, LDLRAD4, SEH1L, DLGAP2, DCTN2, GRID2*, and *KIF5A* among both ASD and ADHD cases (Fig. 2).^10^

Disruptive variants in gene-sets involved in multiple intracellular signaling pathways and DNA instability have been observed previously in ASD.^23^ Variants in gene pathways associated with DNA/“genomic instability” are increased in both ASD and SCZ.^20^ Consistent with these studies, we observed a 2.77-fold higher proportion of cases with NDD-CNVs among those with GIG-CNVs, than among those without.

This study had certain limitations. (i) Most cases, with the exception of SCZ, were recruited as children or adolescents on the basis of a specific diagnosis. ASD and ADHD have early onset, and many participants would not have reached the age for adolescent or adult-onset disorders, including OCD and SCZ. It is possible that individuals with early onset conditions will develop additional later-onset comorbidities. All SCZ cases in the current study were adults. (ii) Recruitment was by clinicians who focus on a single disorder. It is possible that some cases may have had other NDDs, which were not reported. For example, we had data on intellectual disability/IQ for the SCZ cohort and for some cases with other NDDs. We searched the genotype data for possible multiple ascertainment of any case and found no examples of subjects that were recruited through multiple disorders. We examined for non-primary phenotypes for specific cases with variants in NDD-relevant genes. (iii) Due to limitations of the technology, we studied CNVs only of a certain size (>20 kb) for the majority of samples where we did not have sequence data. Smaller CNVs and single nucleotide polymorphisms also contribute to the etiology of NDDs,^8,35^ but these would have been missed. A more sensitive technology such as genome sequencing would allow more comprehensive detection of all relevant variants.^8,44^ The dataset also needs to be analyzed iteratively as more data and better analysis tools become available.^45^

In summary, we highlighted clinically relevant CNVs found through microarray data for ASD, ADHD, OCD, and SCZ. We also demonstrated that identical CNVs or genes could potentially contribute to the etiology of multiple NDDs, consistent with previous reports,^10,20,46,47^ and providing a valuable resource for comparison in other studies.

## Methods

### Samples

This project was a part of a multilateral collaborative project to investigate genetic etiology across four neurodevelopmental disorders: ADHD, ASD, OCD, and SCZ. This study was approved by the Research Ethics Board at The Hospital for Sick Children. A written informed consent was obtained from all participants or substitute decision makers. CNVs were detected on the same high-resolution microarray platform. The criteria for meeting a diagnosis of ASD, ADHD, OCD, or SCZ were detailed in our previous publications^8,10,13,23,24^ with a few modifications for ADHD (see Supplementary Information). Data from all OCD individuals and 139/435 (32%) of the SCZ cohort had been previously published,^11,13^ but we included them here for comparative purposes (Supplementary Information). ADHD and ASD samples were not previously published. Additional supportive evidence for cross-disorder associations of selected CNVs came from our previously published schizophrenia cohorts^11,24^ and an additional ADHD cohort^10^; all were genotyped on the Affymetrix SNP 6.0 microarray (Table 3).

### Genotyping and detection of rare variants

We extracted genomic DNA from saliva or blood and genotyped samples on the Affymetrix CytoScan HD platform. Quality control and ancestry assessment procedures were as discussed previously.^25^ Using PLINK v1.90b2, we found 1,995 (74.1%) of cases to be of European ancestry (Supplementary Table 1A).

CNVs were identified as previously described.^13,25^ Briefly, four different algorithms were used to call high-confidence CNVs. These included the Affymetrix Chromosome Analysis Suite, iPattern, BioDiscovery Nexus, and Partek Genomics Suite. We defined a stringent set of variants of at least 20 kb wherein each was identified by at least two algorithms and spanned by at least five consecutive probes (Supplementary Table 1B). We defined rare CNVs as those present at no more than 0.1% frequency among 10,851 controls samples (detailed in Zarrei et al.^25^). We further restricted our list to those with more than 75% overlap with copy-number stable regions, according to our stringent CNV map of the human genome.^2^ We confirmed clinically relevant CNVs (Tables 1, 2, Supplementary Table 1C) (as defined below) using a SYBR® Green-based real-time quantitative PCR assays, TaqMan® copy number assays or whole genome sequencing data (if available). The genomic coordinates used are based on Human Genome Build GRCh37/hg19.

### Prioritizing variants relevant to NDDs and the NDD gene list

To focus on CNVs relevant to NDDs, we first selected those variants coinciding with known recurrent genomic disorders, aneuploidies, and large (>3 Mb) deletions and duplications. We also analyzed whether rare CNVs in our cases were similar to those in clinically relevant CNV databases at The Department of Paediatric Laboratory Medicine, The Hospital for Sick Children, comprising over 20,000 cases. We classified variants for their clinical impact according to American College of Medical Genetics guidelines.^26^ Our prioritized variants also included those impacting the coding sequences of genes with sufficient evidence for being clinically relevant to NDDs (Supplementary Information).

### Cross-disorder gene discovery and genes in more than one case in a single disorder

We searched for genes that were impacted by CNVs of 20 kb to 3 Mb in more than one case. Of these, we analyzed brain-expressed genes^48^ that were impacted by rare deletions and that are moderately to strongly constrained in the general population for LoF variants (as defined by a LoF probability of > 0.45^49^; *n* = 1,116). We also analyzed genes whose full transcript length was impacted by duplication.

### Global burden test for protein-coding genes and lncRNAs

We performed a univariate analysis to test the global burden of variants impacting coding sequences of protein-coding genes and all exons of lncRNAs using a logistic regression model. We further tested a burden for brain-expressed protein-coding genes (*n* = 3,666) and lncRNAs (*n* = 1,070) to compare with those not expressed in the brain. Chromatin states from the Roadmap Epigenomics Consortium^50^ were used to identify brain-expressed genes (Supplementary Information). We defined controls as parents of cases in the regression analysis. We used sex and the first three principal components of population stratification calculated using PLINK as covariates. The model was also corrected for the total length of CNVs. Finally, we performed a multivariate analysis to investigate whether the burden signals were from the same sets of CNVs as in the univariate analysis (details in Supplementary Information). We considered *p* < 0.05 as statistically significant. We also reported 0.05 < *p* < 0.1 as nearly significant.

### Genomic instability and fragile sites

Replication stress can lead to CNV formation, and fragile sites. A recent study using genome-wide CNVs^20^ demonstrated a link between DNA/genomic integrity and ASD and SCZ. However, using a larger sample size than the current study (1,108 ASD and 2,458 SCZ), they were unable to find pathways enriched in ASD versus SCZ and vice versa. Given smaller cohorts, we performed our analyses in a combined set of all four NDDs to achieve an acceptable statistical power. We first investigated CNVs impacting the coding sequences of genomic instability genes, looking for change in the proportion of cases with rare CNVs in these genes, compared with that of controls. The genomic instability genes comprised 958 protein coding genes identified from the AmiGO database^36^ by searching for the following terms: DNA repair, DNA replication, genome maintenance, DNA damage, and DNA integrity. We also tested for the overall number of rare CNVs and total length of rare CNVs. We then considered whether cases with perturbed genomic instability genes had a different burden of rare CNVs in NDD genes compared to that in cases with intact instability genes.

### Reporting summary

Further information on experimental design is available in the Nature Research Reporting Summary linked to this paper.

## Supplementary information

Supplementary Notes

Reporting Summary Checklist

Supplementary Data 1

## Data Availability

Relevant microarray data are deposited in the database of Genotypes and Phenotypes (https://www.ncbi.nlm.nih.gov/gap/; ID:phs001881.v1.p1). The relevant CNVs are available in dbVar (ID:nstd173).
