# Supplementary material for: A large data resource of genomic copy number variation across neurodevelopmental disorders
Source: NPJ Genom Med. 2019 Oct 7;4:26. doi: 10.1038/s41525-019-0098-3 (PMC6779875; doi:10.1038/s41525-019-0098-3)
Supplement: Supplementary file 1 — Supplementary Notes [file 41525_2019_98_MOESM1_ESM.pdf]

Supplementary Information for: A large data resource of genomic copy number variation across neurodevelopmental disorders

Mehdi Zarrei[1,2], Christie L. Burton[3], Worrawat Engchuan[1,2], Edwin J. Young[4], Edward J. Higginbotham[1,2,5], Jeffrey R. MacDonald[1], Brett Trost[1,2], Ada J. S. Chan[1,2,5], Susan Walker[1], Sylvia Lamoureux[1], Tracy Heung[6], Bahareh A. Mojarad[2], Barbara Kellam[1], Tara Paton[1], Muhammad Faheem[1,2], Karin Miron[1,2], Chao Lu[1], Ting Wang[1], Kozue Samler[1], Xiaolin Wang[1], Gregory Costain[7,8], Ny Hoang[2,5,9], Giovanna Pellecchia[1], John Wei[1], Rohan V. Patel[1], Bhooma Thiruvahindrapuram[1], Maian Roifman[7,10,11], Daniele Merico[1,12], Tara Goodale[3], Irene Drmic[13], Marsha Speevak[14], Jennifer L. Howe[1], Ryan K. C. Yuen[1,2], Janet A. Buchanan[1], Jacob A. S. Vorstman[15,16], Christian R. Marshall[1,4,17], Richard F. Wintle[1], David R. Rosenberg[18,19], Gregory L. Hanna[20], Marc Woodbury-Smith[1,21], Cheryl Cytrynbaum[2,5,7,22], Lonnie Zwaigenbaum[23], Mayada Elsabbagh[24], Janine Flanagan[11], Bridget A. Fernandez[25], Melissa T. Carter[26], Peter Szatmari[15,27,28], Wendy Roberts[16], Jason Lerch[29,30], Xudong Liu[31], Rob Nicolson[32,33], Stelios Georgiades[34], Rosanna Weksberg[2,7,5], Paul D. Arnold[2,35,36], Anne S. Bassett[6,15,37], Jennifer Crosbie[3,15], Russell Schachar[3,15,38], Dimitri J. Stavropoulos[4], Evdokia Anagnostou[39], Stephen W. Scherer[1,2,5,40]

[1]The Centre for Applied Genomics, The Hospital for Sick Children, Toronto, ON, Canada; [2]Program in Genetics and Genome Biology, The Hospital for Sick Children, Toronto, ON, Canada; [3]Neurosciences and Mental Health Program, The Hospital for Sick Children, Toronto, ON, Canada; [4]Genome Diagnostics, Department of Paediatric Laboratory Medicine, The Hospital for Sick Children, Toronto, ON, Canada; [5]Department of Molecular Genetics, University of Toronto, Toronto, ON, Canada; [6]Clinical Genetics Research Program, Centre for Addiction and Mental Health, Toronto, ON, Canada; [7]Division of Clinical and Metabolic Genetics, The Hospital for Sick Children, Toronto, ON, Canada; [8]Medical Genetics Residency Training Program, University of Toronto, Toronto, ON, Canada; [9]Department of Genetic Counselling, The Hospital for Sick Children, Toronto, ON, Canada; [10]The Prenatal Diagnosis and Medical Genetics Program, Department of Obstetrics and Gynecology, Mount Sinai Hospital, Toronto, ON, Canada; [11]Department of Paediatrics, University of Toronto, Toronto, ON, Canada; [12]Deep Genomics Inc., Toronto, ON, Canada; [13]Hamilton Health Sciences, Ron Joyce Children's Health Centre, Hamilton, On, Canada; [14]Trillium Health Partners Credit Valley Site, Mississauga, Ontario, Canada; [15]Department of Psychiatry, University of Toronto, Toronto, ON, Canada; [16]Autism Research Unit, The Hospital for Sick Children, Toronto, ON, Canada; [17]Laboratory Medicine and Pathobiology, University of Toronto, Toronto, ON, Canada; [18]Department of Psychiatry and Behavioral Neurosciences, Wayne State University, Detroit, MI, United States; [19]The Children's Hospital of Michigan, Detroit, MI, United States; [20]Department of Psychiatry, University of Michigan, Ann Arbor, MI, United States; [21]Institute of Neuroscience, Newcastle University, Newcastle upon Tyne, United Kingdom; [22]Dalla Lana School of Public Health and the Department of Family and Community Medicine, University of Toronto, Toronto, ON, Canada; [23]Department of Pediatrics, University of Alberta, Edmonton, AB, Canada; [24]Montreal Neurological Institute, McGill University, Montreal, QC, Canada; [25]Discipline of Genetics, Faculty of Medicine, Memorial University of Newfoundland, St. John’s, NL, Canada; [26]Regional Genetics Program, The Children’s Hospital of Eastern Ontario,

Ottawa, ON, Canada; [27]Centre for Addiction and Mental Health, Toronto, ON, Canada; [28]Department of Psychiatry, The Hospital for Sick Children, Toronto, ON, Canada; [29]Mouse Imaging Centre, Hospital for Sick Children, Toronto, ON, Canada; [30]Department of Medical Biophysics, The University of Toronto, Toronto, ON, Canada; [31]Department of Psychiatry, Queen's University, Kinston, ON, Canada; [32]Children's Health Research Institute, London, ON, Canada; [33]Western University, London, ON, Canada; [34]Department of Psychiatry and Behavioural Neurosciences, McMaster University, Hamilton, ON, Canada; [35]Mathison Centre for Mental Health Research and Education, University of Calgary, Calgary, AB, Canada; [36]Departments of Psychiatry and Medical Genetics, Cumming School of Medicine, University of Calgary, Calgary, AB, Canada; [37]The Dalglish Family 22q Clinic, Toronto General Hospital, Toronto, ON, Canada; [38]Institute of Medical Science, University of Toronto, Toronto, ON, Canada; [39]Holland Bloorview Kids Rehabilitation Hospital, University of Toronto, Toronto, ON, Canada; [40]Department of Molecular Genetics and McLaughlin Centre, University of Toronto, Toronto, ON, Canada

Correspondence: Stephen W. Scherer (stephen.scherer@sickkids.ca)

Supplementary Methods: Neurodevelopmental disorders (NDD) diagnostic criteria

The Parent Interview for Child Symptoms (PICS)[1] is similar to the Schedule for Affective Disorders and Schizophrenia (KSADS)[2] with an enhanced module for attention deficit hyperactivity disorder (ADHD) and other disruptive behavior disorders. A clinical psychologist conducted the PICS. Reliability of ADHD diagnoses was assessed through videotaped interviews in 48 cases and was found to be high (interclass correlation for total symptom score = 0.93). Teacher information was collected using the Child Behavior Checklist Teacher form and the Strengths and Weaknesses of ADHD Symptoms and Normal Behavior (SWAN) teacher form. A clinical psychologist assessed intelligence and academic attainment.

To receive a best-estimate diagnosis of ADHD, arrived at through consensus between the assessing psychiatrist and psychologist, the participant had to present with impairing and developmentally atypical symptoms before age 7, meet Diagnostic and Statistical Manual of Mental Disorders, Fourth Edition (DSM-IV) criteria based on PICS and/or Teacher CBCL, exhibit evidence of symptoms and impairment both at home and at school, and not present with any of the exclusion criteria for ADHD as stated in DSM-IV. Individuals with full-scale IQ data were excluded if they had an IQ of less than 80 on both the verbal and the performance subscales of the Wechsler Intelligence Scale for Children. The mean full-scale IQ for these subjects was 100.32 (SD = 13.87; n = 174).

An autism spectrum disorder (ASD) diagnosis was of research quality when it met criteria on one or both of the diagnostic measures, Autism Diagnostic Interview–Revised and Autism Diagnostic Observation Schedule; it was considered a clinical diagnosis when given by an expert clinician according to DSM-IV or 5.[3,4]

Ascertainment and phenotyping of the schizophrenia (SCZ) cohort was as described in detail elsewhere.[5,6] In brief, were ascertained adult patients who met the DSM-IV diagnostic criteria for schizophrenia or schizoaffective disorder from community mental health clinics in Central and

Eastern Canada. The study was approved by local hospital and university institutional review boards and written informed consent was obtained for all participants. All study participants underwent direct clinical screening assessments for potential syndromic features using a standardized protocol that included review of available lifetime medical records and assessment of physical features.[7] All phenotyping was done blind to genotype. In addition to the ascertainment as above, the current study included five adults with schizophrenia referred by community psychiatrists for 22q11.2 deletion syndrome as described elsewhere.[8] Of the n=204 with SCZ studied (from 200 families), 31 (16.9%) with sufficient data on cognitive functioning had ID, including three with severe ID. Of the 204, 139 (68.1%) were previously published.[6] Six of the 204 had typical 22q11.2 deletions, five of whom were referred with syndromic features and previously published.[8]

For obsessive-compulsive disorder (OCD), we interviewed participants and their parents with the Schedule for Schizophrenia and Affective Disorders for School-Aged Children-Present and Lifetime Version.[9] In addition, we used the Schedule for Obsessive-Compulsive and Other Behavioral Syndromes [10] at the University of Michigan and Wayne State sites. We assessed specific symptoms and current severity of OCD in the participants using the Children's Yale-Brown Obsessive Compulsive Disorder Scale.[11] The site clinical investigator - a child and adolescent psychiatrist - made lifetime and current axis one diagnoses using all sources of information according to DSM-IV criteria.

Supplementary Methods: NDD genes

Our NDD gene list (Supplementary Table 1G) included those expertly annotated for association with ASD or intellectual disability, and high confidence or statistically significant genes from NDD sequencing studies. These reference sources included: 1) Tier 1 and 2 high confidence developmental brain disorder genes (each with at least two de novo LOF variants)[12] 2) 61 high confidence ASD genes from the MSSNG whole-genome sequencing project[3] 3) 65 ASD candidate genes (false discovery rate ≤ 0.1) identified from Simons Simplex Collection and Autism Sequencing Consortium exome sequencing data[13] 4) 10 novel candidate genes for intellectual disability (Benjamini-Hochberg corrected *P*-value <0.05)[14] 5) 94 genes with increased numbers of damaging de novo variants ($p < 7 \times 10^{-7}$) from Deciphering Developmental Disorders study[15] 6) genes with increased *de novo* variants in epileptic encephalopathy[15] intellectual disability/epilepsy[16] or schizophrenia cohorts[17,18] 7) 346 genes with haploinsufficiency or triplosensivity scores of 1 to 3 from the ClinGen Dosage Sensitivity Map[19] and 8) SFARI autism candidate genes (n=260) with associated scores ranging from 1 to 3 (https://www.sfari.org/).

Supplementary Methods: Burden analysis of CNVs impacting brain expressed protein coding and lncRNA genes

We applied a logistic regression test for the burden of protein coding and lncRNA genes impacted by rare CNVs, in cases compare to controls (parents or unaffected individuals). Only rare CNVs (< 0.1% frequency) larger than 20kb were tested. We corrected for the distribution of CNVs in different sexes and sub-populations using sex and population stratification by principal component analysis (PCA). We also corrected for the difference in number of rare CNVs among samples by total size of rare CNVs having excluded centromeres, telomeres and segmental duplications. We independently tested the burden of deletions and duplications in exons of

protein coding and lncRNA genes. The formula of the logistic regression model is shown in below, where M0 and M1 models were compared by *anova* function of the R package to derive *p*-value of the test.

M0: $case/control\ status = \beta_1 \times sex + \beta_2 \times PC1 + \beta_3 \times PC2 + \beta_4 \times PC3 + \beta_5 \times clean\_size + \varepsilon$

M1: $case/control\ status = \beta_1 \times sex + \beta_2 \times PC1 + \beta_3 \times PC2 + \beta_4 \times PC3 + \beta_5 \times clean\_size + \beta_6 \times no\_genes + \varepsilon$

where $\beta_{[1\text{-}6]}$ are beta coefficients, *sex* is sex of sample, *PC[1-3]* are principal components from population stratification, *clean_*size is total size of rare CNVs and *no_genes* is number of protein coding or lncRNAs impacted by rare CNVs.

Besides the burden testing of all genes, we tested the brain-expressed genes independently. Due to the lack of expression data of lncRNAs, we used the chromatin states data from Roadmap epigenomics[20] to define the brain-expressed regions of the genome. The chromatin states data contained data from 48 non-brain tissues and 10 brain tissues. We only focused on analysing regions defined as a transcribed state (3_TxFlnk, 4_Tx or 5_TxWk). To be more specific to brain, the brain-expressed regions were those with no transcribed state in any of non-brain tissues, or an odd ratio of brain tissues over non-brain tissues is >5. We defined a brain-expressed gene as one that had exonic overlap with brain-expressed regions. We corrected for multiple testing using permutation-based FDR, as some tests are correlated with each other. We performed 1,000x of label permutation using BiasedUrn library of R package.[21] The permutation-based FDR was calculated as the ratio of permutated tests that passed the given p-value threshold over the ratio of actual tests that passed the same p-value threshold. Finally, we did a multivariate analysis to test whether signals from brain-expressed protein coding and lncRNAs were in common. The formula of logistic regression for multivariate analysis is:

M0: $case/control\ status = \beta_1 \times sex + \beta_2 \times PC1 + \beta_3 \times PC2 + \beta_4 \times PC3 + \beta_5 \times clean\_size + \varepsilon$

M1: $case/control\ status = \beta_1 \times sex + \beta_2 \times PC1 + \beta_3 \times PC2 + \beta_4 \times PC3 + \beta_5 \times clean\_size + \beta_6 \times no\_lncRNA\_genes + \beta_7 \times no\_protein\_coding\_genes + \varepsilon$

Supplementary Methods: Burden analysis of CNVs impacting NDD genes in case with rare CNVs impacting genomic instability genes

We compiled 958 protein coding genes involved in genomic instability from the AmiGO database.[22] We tested for the burden of genomic instability genes impacted by either deletions or duplications between cases and controls using brain-expressed genes. We performed the same burden correction for sex, population stratification and total size of rare CNVs. We compared the number of CNVs and total size of CNVs between samples with and without rare CNVs impacting genomic instability genes, using a simple Welch two sample t-test. Additionally, we compiled a list of 1,160 genes associated with NDD phenotypes for the last set of tests to answer whether cases with CNVs impacting genomic instability genes tend to have CNVs impacting NDD genes as well. The test was done using Fisher’s exact test.

Supplementary Data 1: **Supplementary Tables 1A-G** [see online Supplementary Data 1.xlxs]

Supplementary References

1 Ickowicz, A. *et al.* The parent interview for child symptoms: a situation-specific clinical research interview for attention-deficit hyperactivity and related disorders. *Can J Psychiatry* **51**, 325-328, (2006).

2 Ambrosini, P. J. Historical development and present status of the schedule for affective disorders and schizophrenia for school-age children (K-SADS). *J Am Acad Child Adolesc Psychiatry* **39**, 49-58, (2000).

3 Yuen, R. K. *et al.* Whole genome sequencing resource identifies 18 new candidate genes for autism spectrum disorder. *Nat Neurosci* **20**, 602-611, (2017).

4 Woodbury-Smith, M. *et al.* A genome-wide linkage study of autism spectrum disorder and the broad autism phenotype in extended pedigrees. *J Neurodev Disord* **10**, 20, (2018).

5 Costain, G. *et al.* Pathogenic rare copy number variants in community-based schizophrenia suggest a potential role for clinical microarrays. *Hum Mol Genet* **22**, 4485-4501, (2013).

6 Lowther, C. *et al.* Impact of IQ on the diagnostic yield of chromosomal microarray in a community sample of adults with schizophrenia. *Genome Med* **9**, 105, (2017).

7 Bassett, A. S. *et al.* Clinically detectable copy number variations in a Canadian catchment population of schizophrenia. *J Psychiatr Res* **44**, 1005-1009, (2010).

8 Van, L. *et al.* All-cause mortality and survival in adults with 22q11.2 deletion syndrome. *Genet Med*, (2019).

9 Kaufman, J. *et al.* Schedule for Affective Disorders and Schizophrenia for School-Age Children-Present and Lifetime Version (K-SADS-PL): initial reliability and validity data. *Journal of the American Academy of Child and Adolescent Psychiatry* **36**, 980-988, (1997).

10 Hanna, G. L. *Schedule for Obsessive-Compulsive and Other Behavioral Syndromes.* (University of Michigan, Ann Arbor, MI, 2007).

11 Scahill, L. *et al.* Children's Yale-Brown Obsessive Compulsive Scale: reliability and validity. *Journal of the American Academy of Child and Adolescent Psychiatry* **36**, 844-852, (1997).

12 Gonzalez-Mantilla, A. J., Moreno-De-Luca, A., Ledbetter, D. H. & Martin, C. L. A Cross-Disorder Method to Identify Novel Candidate Genes for Developmental Brain Disorders. *JAMA Psychiatry* **73**, 275-283, (2016).

13 Sanders, S. J. *et al.* Insights into Autism Spectrum Disorder Genomic Architecture and Biology from 71 Risk Loci. *Neuron* **87**, 1215-1233, (2015).

14 Lelieveld, S. H. *et al.* Meta-analysis of 2,104 trios provides support for 10 new genes for intellectual disability. *Nat Neurosci* **19**, 1194-1196, (2016).

15 Deciphering Developmental Disorders, S. Prevalence and architecture of de novo mutations in developmental disorders. *Nature* **542**, 433-438, (2017).

16 Halvardson, J. *et al.* Mutations in HECW2 are associated with intellectual disability and epilepsy. *J Med Genet* **53**, 697-704, (2016).

17 Xu, B. *et al.* De novo gene mutations highlight patterns of genetic and neural complexity in schizophrenia. *Nat Genet* **44**, 1365-1369, (2012).

18 Fromer, M. *et al.* De novo mutations in schizophrenia implicate synaptic networks. *Nature* **506**, 179-184, (2014).
19 Rehm, H. L. *et al.* ClinGen--the Clinical Genome Resource. *N Engl J Med* **372**, 2235-2242, (2015).
20 Roadmap Epigenomics, C. *et al.* Integrative analysis of 111 reference human epigenomes. *Nature* **518**, 317-330, (2015).
21 Epstein, M. P. *et al.* A permutation procedure to correct for confounders in case-control studies, including tests of rare variation. *Am J Hum Genet* **91**, 215-223, (2012).
22 Carbon, S. *et al.* AmiGO: online access to ontology and annotation data. *Bioinformatics* **25**, 288-289, (2009).
